# Puerperal Eclampsia

**Published:** 1878-06

**Authors:** W. B. Prather

**Affiliations:** Florence, Ga.


ATLANTA
Medical and Surgical Journal.

VOL. XVI.] JUNE—1878. [No. 3

### Original Communications.

### PUERPERAL ECLAMPSIA.

BY W. B. PRATHER, M.D., FLORENCE, GA.

For the last year or two I have seen several communications in reference to the treatment of puerperal eclampsia, and having had several cases to treat myself in the last few years, thought I would contribute this, my mite, and if you thought it worth mentioning in the JOURNAL, you are privileged to do so.

On the night of the 4th inst., was called to see Fanny T., (colored), who was about six and a half months advanced in her eighth pregnancy. Upon enquiring, I learned for several days previous to my being called in she had complained of headache, was drowsy and generally indisposed. Feet and legs presented a dropsical appearance, and face puffed.

I had not been in the room more than five minutes when she was seized with a convulsion of the hardest kind. I corded the arm and took by guess twenty or twenty-five ounces of blood, and used chloroform inhalations, veratrum (Norwood's) and morphine hypodermically. This lessened the severity of the convulsions somewhat, and as soon as the patient could be induced to swallow, I gave 15 grains calomel every two hours until four doses were taken. I remained with the patient until the following evening, when, by the use of ergot, she was delivered of a male child, dead probably two or three days. The convulsions in this case lasted at lengthened intervals for twenty hours, but had only two light ones after the birth of her child. During all this time the bowels had not responded to the calomel; I therefore ordered an enema of warm water and common salt, which effectually moved the bowels. From this time forward she gradually improved until the end of the third day she regained consciousness, and at the present writing the recovery is complete, with the exception of weakness, which time will remedy.

This case was treated on the same general plan with four others, with recovery of all but one case, death taking place in thirty minutes after my arrival.

I will state here that I was induced to use the hypodermic veratrum by seeing mention made of it in the JOURNAL by Dr. J. W. Griggs, of West Point, Ga.

In conclusion, I would say, that a judicious use of the lancet, followed up by inhalations of chloroform, hypodermic veratrum and morphia, with large doses of calomel, constitutes the best method of treatment.

